# T cell receptor-Vβ repertoires in lung and blood CD4^+^ and CD8^+^ T cells of pulmonary sarcoidosis patients

**DOI:** 10.1186/1471-2466-14-50

**Published:** 2014-03-22

**Authors:** Kerstin M Ahlgren, Tina Ruckdeschel, Anders Eklund, Jan Wahlström, Johan Grunewald

**Affiliations:** 1 Respiratory Medicine Unit, Department of Medicine, Solna and Center for Molecular Medicine, Karolinska Institutet and Karolinska University Hospital Solna, Stockholm, Sweden

**Keywords:** T cell, T cell receptor, Sarcoidosis, Bronchoalveolar Lavage, Vβ-repertoire, flow cytometry

## Abstract

**Background:**

Sarcoidosis patients have accumulations of activated CD4^+^ T cells in affected organs, such as the lungs. T cell receptor (TCR) Vβ-chain usage has been incompletely characterized in these patients**.**

**Methods:**

We surveyed the TCR Vβ usage in CD4^+^ and CD8^+^ T cells in bronchoalveolar lavage (BAL) cells and peripheral blood mononuclear cells (PBMC) from 15 HLA-typed Scandinavian sarcoidosis patients. In addition, PBMC from 9 healthy volunteers and BAL cells from three of them were examined. Using 21 Vβ family-specific antibodies, we covered approximately 70% of all Vβ chains.

**Results:**

In BAL T cells from sarcoidosis patients, we identified 16 CD4^+^ T cell expansions in 271 analyses (5.9%) and 21 CD8^+^ expansions in 240 analyses (8.7%). In PBMC we found 9 CD4^+^ expansions in 276 analyses (3.3%) and 12 CD8^+^ expansions out of 263 analyses (4.6%). Consistent with previous studies we found Vβ8 and Vβ16 expansions in sarcoidosis patients’ lungs. In addition, we found lung restricted Vβ22 expansions in three HLA DRB1 03^+^ patients. However, we found no statistically significant difference in frequency of expansions between patients and healthy controls.

**Conclusions:**

The identified T cell expansions in present study indicate specific antigen recognition in the lungs of sarcoidosis patients.

## Background

Sarcoidosis is a multisystem disorder characterized by an accumulation of activated CD4^+^ T cells in the affected organs [1]. In the majority of cases the lungs are targeted by disease. Many of the patients suffer from respiratory symptoms such as dyspnea, dry cough and chest pain. Bronchoscopy with bronchoalveolar lavage (BAL) is commonly performed to examine patients exhibiting respiratory symptoms and clinical signs of sarcoidosis. BAL can also be used as a research tool because the aspirate includes cells most likely relevant for the pathophysiology [2]. From studies of BAL cells we know that lung accumulated immune cells are activated in sarcoidosis patients [3,4], further, accumulations of CD4^+^ cells expressing the T cell receptor (TCR) V gene segment Vα2.3^+^ are accumulated in BAL fluid (BALF) of HLA DRB1*03^+^ patients, often with Löfgren’s syndrome [5].

Characterizations of TCR gene usage in lung and blood of patients can provide insights into disease associated processes [6-9]. T cell recognition of a particular antigen presented by an HLA molecule can result in a clonal expansion of T cells bearing identical TCRs. The majority of T cells express the αβ TCR. The variable (V) region of the β-chain is generated by recombination through somatic rearrangement of one V region gene to a D and a J segment selected from a pool of discontinuous gene segments. By RNA splicing the VDJ segment is put together with one of the two C region genes. A similar principle applies to the α-chain, consisting of V and J segments. The TCR V region genes are not randomly used within CD4^+^ and CD8^+^ T cells. There are several V α and β genes that have a significant skewing to either CD4^+^ or CD8^+^ cells [10,11]. Most likely this is due to interactions of the V region gene products with MHC II molecules (for CD4^+^ skewing), or MHC I (for CD8^+^), during thymic maturation of the T cells. Hence, we analyzed these cell subsets separately. Previous studies have assessed the TCR α and β chain gene segments in sarcoidosis, mainly by PCR amplification [5,12-14].

The etiology of sarcoidosis is still not clear. Most evidence supports a genesis including a trigger by one or more still unknown antigens in the lungs and an aberrant immune response in genetically susceptible individuals. We hypothesise that antigenic triggering in the lungs of patients would give rise to T cell clones expressing certain Vβ-segments at higher frequency compared to the corresponding frequency in blood.

In the present study we used flow cytometric analysis of T cells stained with a large panel of 24 TCR Vβ specific antibodies, covering about 70% of the normal TCR Vβ repertoire in both CD4^+^ and CD8^+^ T cells. We mapped the Vβ repertoire in BALF cells and peripheral blood mononuclear cells from 15 patients with pulmonary sarcoidosis, six of them with Löfgren’s syndrome.

## Methods

### Study subjects

BALF samples, and peripheral blood from all patients but one, were obtained from 15 Scandinavian patients (values show median age, with min- max range in parenthesis) [43 (30–74) years old; 7 women] coming to the Respiratory Medicine unit at the Karolinska University Hospital, Stockholm, Sweden, for diagnostic investigation (as outlined in ref [1]). All patients included in the study had an active disease with symptoms such as fatigue, dyspnea on exertion, and dry coughing, as well as findings on chest radiography in line with sarcoidosis. All were investigated clinically and with pulmonary function tests, as well as with bronchoscopy with bronchoalveolar lavage, and were diagnosed with sarcoidosis according to criteria established by World Association of Sarcoidosis and other Granulomatous Disorders (WASOG) [15]. Blood from 9 healthy non-smoking volunteers [35 (18–51) years old; 7 women] and BALF from three of them were included as controls. All subjects gave their informed consent. The study was approved by the Regional Ethical Review Board in Stockholm, Sweden. The clinical characterization is outlined in Table 1. At the time of bronchoscopy none had any systemic anti-inflammatory treatment. All subjects undergoing BAL were HLA-typed.

**Table 1:** Characteristics of the sarcoidosis patients

**Patient characterizations**					**Pulmonary function tests**		
**Patient**	**HLA DRB1 type**	**CD4/CD8 ratio**	**Lymphocytes in BAL (%)**	Chest X-ray stage*	**VC (%)**	**FEV**_**1 **_**(%)**	**DL**_**co **_**(%)**
1	4, 15	17	49	I	93	100	84
2	3	11.1	25	I	76	82	
3	1, 3	1.8	6.6	II	82	81	87
4	NA	10.3	64	I	69	63	58
5	13, 14	11.8	34	II	108	103	90
6	4, 8	8.9	32	I	90	76	73
7	4, 15	7.3	16.8	II		68	
8	1, 13	14.4	14.2	I	80	79	
9	3, 16	11.3	25.6	I	84	89	
10	3, 15	2.2	7	I	96	107	111
11	13, 15	3.6	12.4	III	73	60	93
12	4, 15	7.2	25.6	I	119	124	111
13	15	3	50	II	85	80	
14	4, 7	2.4	7.6	IV	65	62	55
15	4, 8	6	19	III	99	86	78

NA = not analysed, VC = vital capacity (% of reference value), FEV_1_ = forced expiratory volume in one second, DL_co_ = diffusing capacity of the lung for carbon monoxide. *Chest X-ray stages: I) Bilateral hilar lymphadenopathy (BHL) without pulmonary infiltrates, II) BHL with pulmonary infiltrates, III) pulmonary infiltrates without BHL, IV) fibrosis with distortions.

### BAL procedure and handling of cells

BAL was performed under local anaesthesia by an experienced physician as previously described [16]. In short, a flexible fibreoptic bronchoscope was passed transorally and wedged into the middle-lobe bronchus. Sterile phosphate buffered saline at 37°C was instilled in five aliquots of 50 ml and immediately re-aspirated and collected into a siliconised plastic bottle kept on ice. The mean recovery of the instilled fluid was 63% (min-max range 42–73%). BALF was strained through a Dacron net (Millipore, Cork, Ireland) before centrifugation. The cell pellet was resuspended in cold phosphate buffered saline (PBS) pH 7.4, and kept on ice throughout the experiments. BAL fluid differential cell counts were based on May-Grünwald and Giemsa staining (Table 2).

### Peripheral blood mononuclear cells

Whole blood was collected into Sodium Heparinised tubes. Peripheral blood mononuclear cells were isolated using Ficoll Paque PLUS (GE Healthcare, Uppsala, Sweden) according to the manufacturer’s instructions.

### Flow cytometric analysis of Vβ repertoires

BAL and blood cells were stained using the following antibodies; CD3-Pacific Blue (BD Pharmingen), anti CD4-APC-H7, CD8-AmCyan (11 of 15 patient samples and all control samles) (BD Bioscience). Normal mouse serum (Corning Life Science, Tewksbury, MA, USA) was used as Fc-Block. TCR Vβ was stained using an eight-tube panel, containing 24 monoclonal antibodies known to react with specific TCR-Vβ families (IO Test Beta Mark TCR Vβ Repertoire Kit)(Beckman Coulter, Brea, CA, USA). Each test, containing 1 million BAL cells or 0.5 million PBMC, permitted analysis of three Vβ families simultaneously as each of the eight tubes included a mixture of three antibodies conjugated to fluorescein isothiocyanate, phycoerythrin or fluorescein isothiocyanate and phycoerythrin. Due to limitations in cell numbers, all Vβ families were not analysed in all subjects. Nomenclature for the Vβ families used throughout this paper is from [17]. Flow cytometry was run on a BD FACS Canto II. Results were expressed as the percentage of CD4^+^ and CD8^+^ T cells, respectively, that expressed the various TCR Vβ families.

### Definition of T cell expansion and statistical analysis

T cell expansions were defined as in [18], i.e. any expression of a certain Vβ family as percentage of the gated population (CD4^+^ or CD8^+^ T cells) > 2 SD above the reference value was considered a significant T cell expansion. Reference values for the frequency of Vβ gene segment found in CD4^+^ and CD8^+^ blood T cells of healthy subjects (based on a cohort of 85 (or 46 for Vβ4, Vβ7.2 and Vβ13.2) normal blood specimens) were provided by the manufacturer of the test (Beckman Coulter, Brea, CA, USA). The healthy control values in this study were consistent with the reference values, except for Vβ4, Vβ5.3 and Vβ9, which had a very low signal in all samples and Vβ12, which had a higher expression in our CD8^+^ samples compared to reference values on CD8^+^ cells (Additional file 1). Vβ4, Vβ5.3 and Vβ9 were therefore excluded from all further analyses. Since the normal TCR repertoire in BAL fluid has not been extensively studied, the reference values obtained from whole blood were also used for CD4^+^ and CD8^+^ BAL T cells.

Wilcoxons signed ranks test was used to calculate differences in Vβ expression in lung and blood cells. Fisher’s exact test was used to calculate difference in frequency of expansions between groups. Differences with p-values ≤ 0.05 were considered statistically significant.

## Results

### Patient characteristics

Fifteen patients with sarcoidosis were included. The sarcoidosis diagnosis was made based on typical clinical and radiographic manifestations [1] and findings at bronchoscopy with BAL according to criteria as outlined by WASOG [15]. Two patients had ocular sarcoidosis. Six patients with Löfgren's syndrome, with typi cal acute onset of disease and chest X-ray stage I-II were included in the study. Four of them had erythema nodosum; none had ankle arthritis (Table 1). In addition we were able to recruit three healthy controls for BAL.

The majority of patients (73%) had a CD4/CD8 ratio above 3.5 (median 7.3, min-max range 1.8-17), the BALF contained 25% (median, min-max range 6.6-64 lymphocytes) and 8 patients included had chest X-ray stage I; four of the patients stage II, two patients stage III and one stage IV. Five patients were ex-smokers and one was current smoker. Diffusing capacity of carbon monoxide (DLco) had a statistically significant inverse correlation with the number of CD8^+^ T cell expansions in BAL from sarcoidosis patients (r = 0.85, p = 0.002, Spearman correlation). No statistically significant correlation between number of T cell expansions and other pulmonary functions tested (VC and FEV_1_), chest X-ray stage, CD4/CD8 ratio, sex or age were identified (Tables 1 and 2).

**Table 2:** Analysis of the bronchoalveolar lavage fluid

**BALF characteristics**			**Differential cell count**			
	**Percent recovery**	Total cell conc. (*10^**6**^**/L)**^**c**^	**Lymphocytes (%)**^**c**^	**Macrophages (%)**^**c**^	**Neutrophils (%)**^**c**^	**Eosinophils (%)**^**c**^
Sarcoidosis patients^a^	68 (42–73)	153 (35–534)	^d^	74 (30–92)	1.0 (0.2-3.4)	0.0 (0–3.0)
Healthy controls^b^	68 (48–71)	77 (62–106)	10 (5.7-11)	88 (88–89)	1.3 (0.7-5)	0.0 (0–0.4)

^a)^n = 15; ^b)^n = 3; ^c)^median (min- max range) ^d)^see result section and Table 1.

#### Vβ repertoire in CD4^+^ T cells of sarcoidosis patients and healthy controls

**Figure 1:**
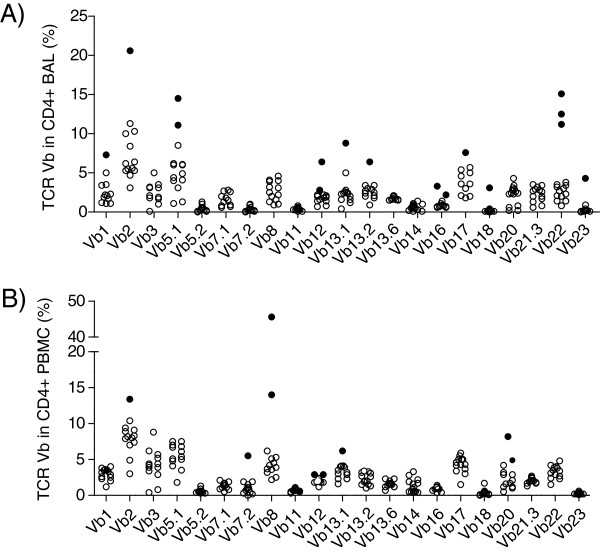
**TCR Vβ expansions in CD4**^**+**^**-BAL (A) and PBMC (B).** Each circle indicates a sample from one patient. Nine of the patients had one or more expansions (filled circles) of CD4^+^ T cells expressing a particular Vβ gene segment in the BAL fluid **(A)**. Vβ5.1, Vβ12 and Vβ16 were expressed as T cell expansions in two patients each, Vβ22 in three patients; Vβ1, Vβ2, Vβ13.1, Vβ13.2, Vβ17, Vβ18 and Vβ23 were expressed as expansions in one patient each. In PBMC **(B)** 6 patients had T cell expansions expressing a particular Vβ gene segment. Vβ2, Vβ7.2 and Vβ13.1 were expressed as expansions in one patient each; segments Vβ8, Vβ12 and Vβ20 were expressed as expansions in two patients each. (Definition of T cell expansion was > mean plus two* SD of a cohort of healthy controls subjects).

To determine whether there is biased expression of TCR Vβ segments in CD4^+^ cells in sarcoidosis, a panel of 24 antibodies covering approximately 70% of the TCR Vβ repertoire was tested on 15 patients. In CD4^+^ BAL cells 16 T cell expansions were identified from 271 analyses (5.9%) (Figure 1A). These T cell expansions appeared in eleven of the 21 studied TCR Vβ chains (Table 3). In CD4^+^ PBMCs, 9 T cell expansions in 6 different Vβ families, were identified of 276 analyses (3.3%) (Figure 1B).

**Table 3:** **TCR V**β **expansions in BAL and PBMC indicating CD4**^**+ **^**or CD8**^**+ **^**expansions and patient and control id with HLA DRB1 type**

**TCR Vb**	**BAL cells**		**Patient/Ctrl**	**HLA**	**PBMC**		**Patient/Ctrl**		**HLA**
Vb1	CD4		P 6	4, 8		CD8	P 1		4, 15
		CD8	P 4						
		CD8	P 5	13, 14					
		CD8	P 9	3, 16					
		CD8	Ctrl 9	4, 15					
Vb2	CD4		P 1	4, 15	CD4		P 5		13, 14
		CD8	P 6	4, 8	CD4		Ctrl 8		12, 15
						CD8	Ctrl 9		4, 15
Vb5.1	CD4		P 13	15					
	CD 4		P 15	4, 8					
Vb5.2						CD8	Ctrl 6		
Vb7.1		CD8	P 14	4, 7					
Vb7.2		CD8	P 2	3	CD4		P 7		4, 15
Vb8		CD8	P 13	15	CD4		P 3		1, 3
		CD8	P 14	4, 7	CD4		P 7		4, 15
Vb11		CD8	P 13	15		CD8	P 3		1, 3
							P 4		P 4
Vb12	CD4		P 8	1, 13	CD4	CD8	P 5		13, 14
	CD4		P 15	4, 8	CD4		P 6		4, 8
		CD8	P 1	4, 15		CD8	P 1		4, 15
		CD8	P 6	4, 8		CD8	P 2		
		CD8	P 12	4, 15	CD4		Ctrl 2		
					CD4		Ctrl 5		
						CD8	Ctrl 1		
						CD8	Ctrl 2		
						CD8	Ctrl 3		
						CD8	Ctrl 5		
						CD8	Ctrl 6		
Vb13.1	CD4		P 13	15	CD4		P 3		1, 3
	CD4		Ctrl 9	4, 15		CD8	P 3		1, 3
							P 5		13, 14
Vb13.2	CD4		P 13	15		CD8	P 13	15	15
		CD8	P 6	4, 8					
	CD4		Ctrl 8						
	CD4		Ctrl 9	4, 15					
Vb13.6		CD8	P 13	15					
	CD4		Ctrl 8	12, 15					
		CD8	Ctrl 7	7, 16					
		CD8	Ctrl 8	12, 15					
Vb16	CD4		P 3	1, 3		CD8	P 1		4, 15
	CD4		P 15	4, 8		CD8	P 2		3
		CD8	P 6	4, 8		CD8	P 5		13, 14
		CD8	P 12	4, 15		CD8	P 11		13, 15
		CD8	P 13	15	CD4		Ctrl 2		
	CD4		Ctrl 7	7, 16	CD4		Ctrl 4		
		CD8	Ctrl 7	7, 16		CD8	Ctrl 2		
		CD8	Ctrl 8	12, 15		CD8	Ctrl 3		
							Ctrl 4		
							Ctrl 9		4, 15
Vb17	CD4		P 15	4, 8		CD8	Ctrl 5		
		CD8	Ctrl 8	12, 15					
Vb18		CD4	P 12	4, 15					
Vb20					CD4		P 1	P 1	4, 15
					CD4		P 13	P 13	15
Vb21.3		CD8	P 2	3					
Vb22	CD4		P 2	3					
	CD4		P 3	1, 3					
	CD4		P 9	3, 16					
		CD8	P 4						
		CD8	P 15	4, 8					
Vb23	CD4		P 3	1, 3					
		CD8	P 3	1, 3					
	CD4		Ctrl 9	4, 15					
	BAL				PBMC				
a	CD4		CD8		CD4			CD8	
Patients	16/271 (5.9%)		21/240 (8.7%)		9/276 (3.3%)			12/263 (4.6%)	
Controls	6/63 (9.5%)		6/63 (9.5%)		5/187 (2.7%)			11/181 (6.1%)	

a) Number of expansions per analysis.

We found two patients with CD4^+^ Vβ8 expansions, one of which contained as many as 45.6% of the CD4^+^ cells. These expansions were not lung restricted, but appeared in the PBMC. In another two patients the Vβ8 segment was expressed as expansions in the lung, however in CD8^+^ T cells. Vβ12 was expressed as expansions in the CD4^+^ population in both lung (two patients) and blood (two patients) (Table 3). Vβ5.1 and Vβ22 CD4^+^ BAL cell expansions were found in two and three patients, respectively (Figure 1A). These expansions seem to be lung restricted.

We identified one TCR Vβ2, two Vβ12 and two Vβ16 expansions in CD4^+^ PBMC of healthy controls (Table 3). In BAL from three healthy controls we identified six expansions in CD4^+^ BAL cells, in 63 analyses (9.5%) (Table 3). These expansions consisted of TCR Vβ13.1, Vβ13.2 (n = 2), Vβ13.6, Vβ16 and Vβ23. We found no significant difference in the frequency of expansions between sarcoidosis patients and controls in CD4^+^ cells (BAL p =0.17; PBMC p = 0.95).

#### Vβ repertoire in CD8^+^ T cells of sarcoidosis patients and healthy controls

**Figure 2:**
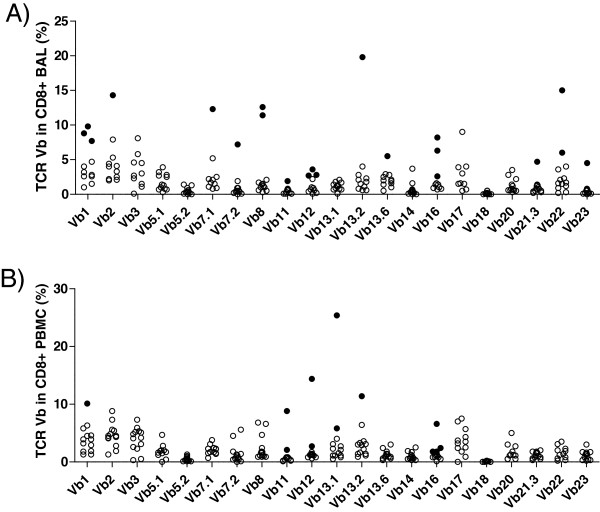
**TCR Vβ expansions in CD8**^**+**^**-BAL (A) and PBMC (B).** Each circle indicates a sample from one patient. Eleven of the patients had one or more expansions (filled circles) of CD8^+^ T cells expressing a particular Vβ gene segment in the BAL fluid. In BAL **(A)** Vβ2, Vβ7.1, Vβ7.2, Vβ11, Vβ13.2, Vβ13.6, Vβ21.3 and Vβ23 were expressed as T cell expansions in one patient each, Vβ8 and Vβ22 in two patients each and Vβ12 and Vβ16 in three patients. In PBMC **(B)** Vβ1 and Vβ13.2 were expressed as expansions in on patient each, Vβ11, Vβ12 and Vβ13.1 in two patients each and Vβ16 in four patients. (Definition of T cell expansion was > mean plus two* SD of a cohort of healthy controls subjects).

The expression of TCR Vβ segments in CD8^+^ cells in sarcoidosis patients was analyzed in the same way as the CD4^+^ cells. There were 21 T cell expansions expressing distinct Vβ segments out of 240 analyses in CD8^+^ BAL cells (8.7%) (Figure 2A) and 12 T cell expansions out of 263 analyses in PBMC (4.6%) (Figure 2B). Three patients had a T cell expansion of cells harboring the Vβ16 segment in CD8^+^ BAL cells. Also in PBMC CD8^+^ cells four Vβ16 T cell expansions appeared, but not in the same individuals as those having Vβ16 expansions in the lung compartment (Table 3). Out of 181 analyses of Vβ usage in CD8^+^ blood cells of healthy control subjects, 12 expansions were identified.

#### Comparisons between lung and blood expression of TCR Vβ in CD4+ and CD8+ cells

**Figure 3:**
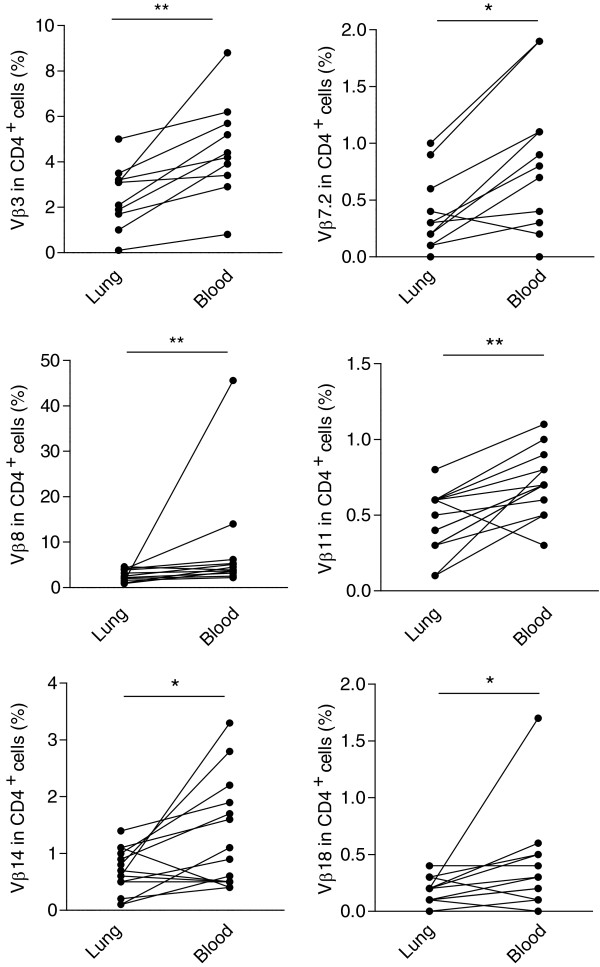
**Differences in Vβ usage between CD4**^**+ **^**T cells in BAL cells and PBMC.** Comparisons between TCR Vβ segment expression in lung and blood revealed a statistically significantly higher expression of segments Vβ3, Vβ7.2, Vβ8, Vβ11, Vβ14 and Vβ18 in blood than lung. (* p <0.05, ** p < 0.01, Wilcoxon’s signed ranks test).

**Figure 4:**
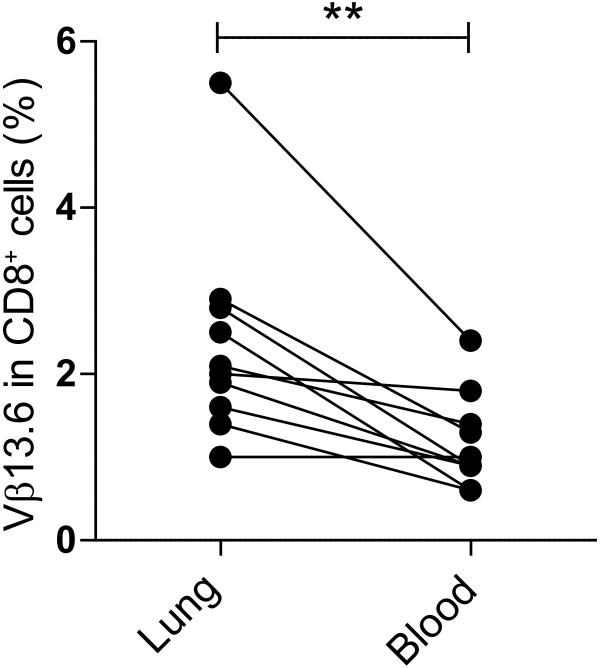
**Higher Vβ13.6 usage in CD8**^**+ **^**BAL cells compared to PBMC.** Comparisons between TCR Vβ segment expression in CD8^+^ T cells in lung and blood revealed a statistically significantly higher expression of segment Vβ13.6 in lung than blood in sarcoidosis patients (** p < 0.01, Wilcoxon’s signed ranks test).

In order to assess whether a bias for TCR Vβ was associated with compartmentalization to the lungs or blood of sarcoidosis patients, we performed a comparison between the Vβ expression in lung and PBMC. There were six Vβ segments with significantly different expressions comparing lung and blood (Figure 3). The segments Vβ3, Vβ7.2, Vβ8, Vβ11, Vβ14 and Vβ18 had a statistically significantly higher (p < 0.05) expression in blood compared to lung. However, we found no segments that had a significantly higher expression in CD4^+^ cells in the lung compartment compared to blood. In CD8^+^ cells, TCR Vβ13.6 has a statistically significantly higher expression in lungs than blood of sarcoidosis patients (p = 0.009) (Figure 4). Comparing the number of T cell expansions, the difference in frequency of CD4^+^ TCR Vβ T cell expansions in lung vs. blood of sarcoidosis patients (p = 0.56) or controls (p = 0.12) was not statistically significant. Neither were there statistical differences in frequency of CD8^+^ TCR Vβ expansions in lung than in blood of sarcoidosis patients (p = 0.15) or controls (p = 0.45).

**Figure 5:**
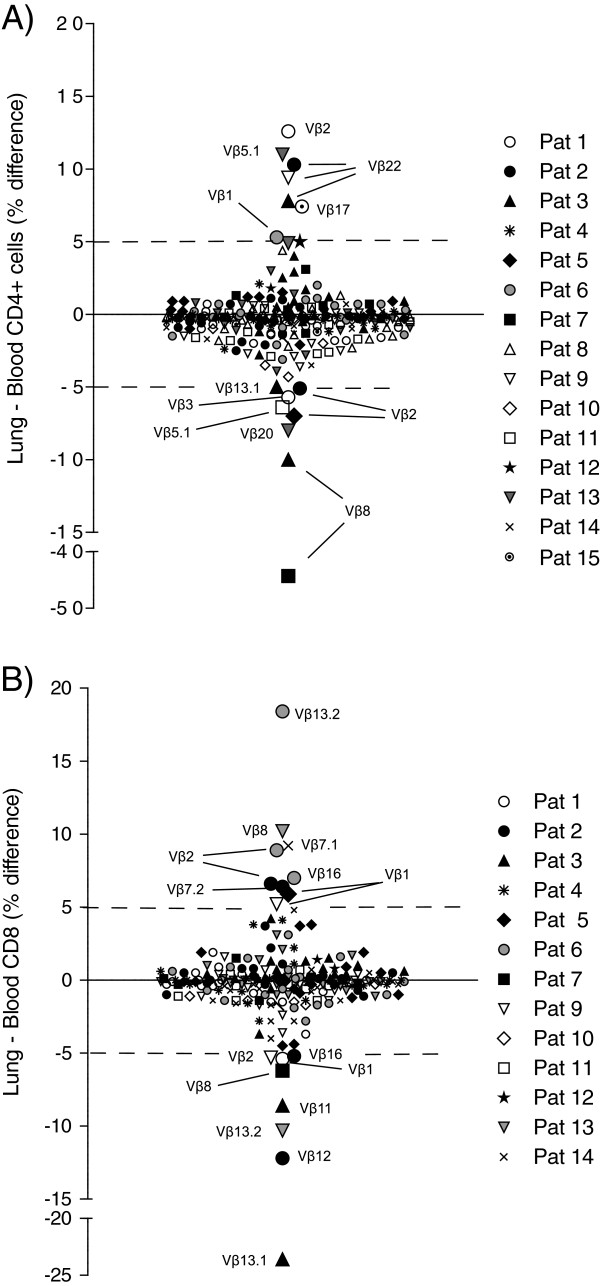
**Vβ segments with absolute usage difference between BAL cells and PBMC.** Each dot indicates difference, BAL vs. PBMC, in one Vβ segments in one patient. The expression (% of the gated population) in PBMC was subtracted from the expression in BAL. Positive values indicate a greater expression in BAL and negative values indicate a greater expression in PBMC. The arbitrary cut off > 5% difference is indicated by a dotted line. **A)** In CD4^+^ T cells 9 Vβ segments had > 5% higher or lower expression in BAL cells versus PBMC in at least one patient. Segments with higher expression in BAL were Vβ1, Vβ17 and Vβ22, while segments Vβ3, Vβ8, Vβ13.1 had higher expression in PBMC. Segment Vβ2 had higher expression in BAL in one patient and higher expression in PBMC in two other patients; Vβ5.1 had higher expression in BAL in one patient and in PBMC in another patient. **B)** In CD8^+^ T cells 10 Vβ segments had > 5% difference in expression comparing BAL cells and PBMC in at least one patient. Vβ1 and Vβ2 both had higher expression in BAL from two patients and in PBMC in one patient. Vβ7.1 and Vβ7.2 had higher expression in BAL in one patient each; Vβ8, Vβ13.2 and Vβ16 had higher expression in BAL in one patient and higher expression in PBMC in another patient each, and the segments Vβ11, Vβ12 and Vβ13.1 had higher expression in PBMC in one patient each.

However, directly comparing the frequencies of accumulated cells with the individual Vβ segments, i.e. without regard to the Vβ usage being classified as an expansion or not, and setting an arbitrary cut off difference between lung and blood compartments of 5%, we identified differences in 8 different segments in the CD4^+^ population (Figure 5A). Three of them appeared in Vβ22. This was also the only segment repeatedly found with a higher value in CD4^+^ BAL cells than PBMC. Segments Vβ2 and Vβ8 had a greater percentage in blood than lung in two patients each. Contrary, one of the other patients (Patient 1) had a higher Vβ2-gene segment expression in the lungs than in blood. In the CD4^+^ T cells, 15 of 244 (6.1%) analyses displayed a greater difference between lung and blood Vβ-gene segment expression than 5%, in any direction. There were 10 different segments in the CD8^+^ cells with differences exceeding 5% in expression between lung and blood (Figure 5B). In CD8^+^ T cells Vβ1 and Vβ2 had a more than 5% higher expression in lung T cells compared to in PBMC in two patients each. Both these segments also had higher expression in blood compared to lung in one patient each. Eight Vβ segments had a higher expression in CD8^+^ PBMC than CD8^+^ lung T cells, and seven had higher expression in lung than blood CD8^+^ cells. These differences did however not appear in more than one patient each. In the CD8^+^ T cells, 17 of 220 (7.7%) analyses displayed greater difference between lung and blood Vβ-gene segment expression than 5%, in any direction.

#### Vβ-repertoire in patients with different disease manifestations and HLA DRB1 alleles

There was no statistically significant difference in frequency of expansions between patients with or without Löfgren’s syndrome, patients with or without erythema nodosum or ocular sarcoidosis, or between CD4^+^ and CD8^+^ cells in sarcoidosis patients (p > 0.05, Fisher’s exact test). 14 of 15 patients were HLA DRB1-typed. In BAL cells, three patients with CD4^+^ Vβ22 expansions were HLA DRB1*03^+^. Two of these three patients had Löfgren’s syndrome. There were three HLA DRB1*04^+^ patients with CD8^+^ Vβ12 expansions. One of these patients had ockular sarcoidosis and one Löfgren’s syndrome with erythema nodosum.

## Discussion

Knowledge about TCR gene segment expression in sarcoidosis can provide insights to the pathogenic process and assist the finding of a potential disease triggering antigen. Previous studies aimed at mapping the TRC Vα and Vβ repertoire has often used PCR technique [5,12-14]. This technique permit analysis of all Vβ gene segments, however not simultaneously in different populations such as CD4^+^ and CD8^+^ cells. The PCR technique does not, in contrast to use of monoclonal antibodies and flow cytometry, measure the expression at the protein level. The latter method is the only method to analyze individual cells Vβ usage. Over time the number of available monoclonal antibodies specific for TCR Vβ family members has increased, now allowing approximately 70% of the TCR Vβ repertoire to be mapped. This led us to the use of monoclonal antibodies directed to TCR Vβ-chain proteins and flow cytometry for the present study. Our major finding was that expansions of T cells expressing Vβ5.1, Vβ7.1, Vβ18, Vβ21.3 and Vβ22 were found only in BAL from sarcoidosis patients. Further, Vβ12 and Vβ16 were the Vβ segments most frequently expressed as expansions in present study. Vβ16 TCR expansion has previously been described to appear in sarcoidosis patients [12].

The patients in our study all had respiratory symptoms, leading to BAL as a part of the clinical examination. This enabled us to study TCR usage in both the lung and blood of the patients. Comparisons between these two compartments in sarcoidosis patients revealed that the expression in the blood, with a few exceptions, exceeded the expression in the lungs. Reference values were provided by the manufacturer of the antibodies. These are based on the Vβ-repertoire in blood lymphocytes from a cohort of healthy subjects, of Mediterranean heritage. In previous studies we have used the definition of a T cell expansion as three times the reference value, or any value above 15% of the gated population. However, since this definition may in fact underestimate T cell expansions we defined a T cell expansion as in a recent study on sporadic inclusion body myositis [18], which defines an expansion as above the reference value plus two SD. Comparing TCR Vβ repertoires in lung and blood, similarities rather than generalized skewing have previously been described [19]. Therefore we used the same reference values for BAL as PBMC. When the total repertoire in each patient was added together and compared to the reference values, we did however note a lower expression in the CD4^+^ BAL cells than in the CD4^+^ PBMCs (data not included). This may indicate a higher normal expression in BAL cells, of certain Vβ segments that is not targeted by the assay in this study.

A higher variability in TCR Vβ expression in CD8^+^ cells than CD4^+^ cells is consistent with previous findings in healthy controls and other diseases [9]. Somewhat surprisingly, we found no significant difference in number of expansions between CD8^+^ cells and CD4^+^ cells or between sarcoidosis patients and controls in present study.

In three patients that were positive for the HLA II allele DRB1*04 we identified TCR Vβ 12 expansions in the CD8+ cell population. However, the CD8 molecule is not interacting with HLA II molecules, but with HLA I molecules. To our knowledge there is no association between HLA DRB1*04 and any HLA class I allele. Vβ12 was the segment most frequently found as T cell expansion in present study. However, our control blood donors displayed a significantly higher Vβ12 expression in CD8^+^ cells than the reference values, suggesting that this may not be a sarcoidosis specific expansion, and may be caused by local deviation from the reference cohort. The reason for this deviation may be the genetic difference between the reference cohort, which is of Mediterranean origin, and our Swedish controls, or due to yet unidentified reasons. The healthy controls in present study were not HLA typed.

Vβ22 expansions were found in CD4^+^ T cells of three patients, all being HLA DRB1*03 positive. These expansions moreover, were lung restricted. HLA DRB1*03 and TCR Vβ22 expansion has previously been described in the context of inflammatory disease, albeit at a lower frequency. In a small study, three out of 9 patients, all three HLA DRB1*03 positive, with Idiopathic Inflammatory Myopathies [6] four Vβ22 T cell expansions were identified. Two of these were located in CD3^+^ cells in muscle, one in CD8^+^ BAL cells and one in CD4^+^ peripheral blood cells.

In a previous report Moller et al. described a biased usage of Vβ8 in sarcoidosis patients’ lung T cells, and to a lesser degree in blood [20]. Consistent with our previous study [13] and a study by Forman et al. [12] in 1994, we noted Vβ8 T cell expansions in BAL in sarcoidosis patients in this study. In the present study we also noted Vβ8 T cell expansions in CD4^+^ PBMC of two sarcoidosis patients.

Vβ5.1 and Vβ22 were the only two Vβ segments appearing exclusively as CD4^+^ T cell expansion in BAL from more than one sarcoidosis patients. Both these segments have been shown to associate with other diseases, such as oral lichen planus and angioimmunoblastic T cell lymphoma [21-23].

We identified CD8^+^ Vβ16 T cell expansions in five sarcoidosis patients, of which three were located in the lung compartment. This TCR expansion has previously been described by Forman *et al.* to appear in sarcoidosis patients [12]. It was not investigated whether these expansions appeared in CD4^+^ or CD8^+^ cells. However, equally to Vβ12 in present study, our healthy controls had a higher expression of Vβ16 than the reference values.

It is known from our previous studies that T cell accumulations tend to withdraw after spontaneous resolution of clinical and radiographic signs of disease in HLA DRB1*03^+^ sarcoidosis patients with Vα2.3 accumulations [3]. Potentially some clonal expansions may already have resolved. The exact disease onset is not possible to determine, but the BAL was in all cases performed as a part of the diagnostic investigation.

## Conclusions

The TCR repertoire is shaped by processes during T cell maturation and exposure to environmental antigens. One pathogen can consist of hundreds of proteins, which each can be broken down to many peptides that are presented in different ways depending on the MHC-molecules on APCs they are presented by. Therefore, even a single specific protein antigen has the potential to give rise to several different Vβ T cell expansions.

Further studies analyzing the T cell responses to candidate antigens and Vβ-repertoire are thus warranted. In the present study we also looked for associations between HLA DRB1 alleles and T cell expansions. However, due to the small sample size only cautious conclusions can be drawn. Further studies could strengthen our conclusion that Vβ8 and Vβ16 T cell expansions are associated to sarcoidosis and Vβ22 associated to the HLA DRB1*03 allele.

Our present results extend the knowledge from the previous studies. This may in the future improve predictions on disease progression and lead to a more individual approach for treatment.

## Abbreviations

TCR: T cell receptor; BAL: Bronchoalveolar lavage; PBMC: Peripheral blood mononuclear cells; BALF: BAL fluid.

## Competing interests

None of the authors has any conflict of interests.

## Authors’ contributions

KMA & TR performed the experiments and analysed the results, AE, JW & JG designed the study, KMA wrote the paper. All authors read and approved the final manuscript.

## Authors’ information

During the study all authors were affiliated at the Karolinska Institutet Lung research laboratory at Karolinska University Hospital, Stockholm, Sweden. AE was also affiliated at the Lung-allergy clinic at the Karolinska University Hospital, Stockholm, Sweden.

## Pre-publication history

The pre-publication history for this paper can be accessed here:

http://www.biomedcentral.com/1471-2466/14/50/prepub

## Supplementary Material

Additional file 1 **The healthy control values in this study were compared with reference values.** The values of our Swedish cohort is consistent with the reference values, except for Vβ4, Vβ5.3 and Vβ9, which had a very low signal in all samples and Vβ12, which had a higher expression in our CD8+ samples compared to reference values on CD8+ cells. Vβ4, Vβ5.3 and Vβ9 were therefore excluded from all further analyses. Since the normal TCR repertoire in BAL fluid has not been extensively studied, the reference values obtained from whole blood were also used for CD4+ and CD8+ BAL T cells. Click here for file
