# Supplementary figures and images for: T cell receptor-Vβ repertoires in lung and blood CD4+ and CD8+ T cells of pulmonary sarcoidosis patients
Source: BMC Pulm Med. 2014 Mar 22;14:50. doi: 10.1186/1471-2466-14-50 (PMC3997965; doi:10.1186/1471-2466-14-50)

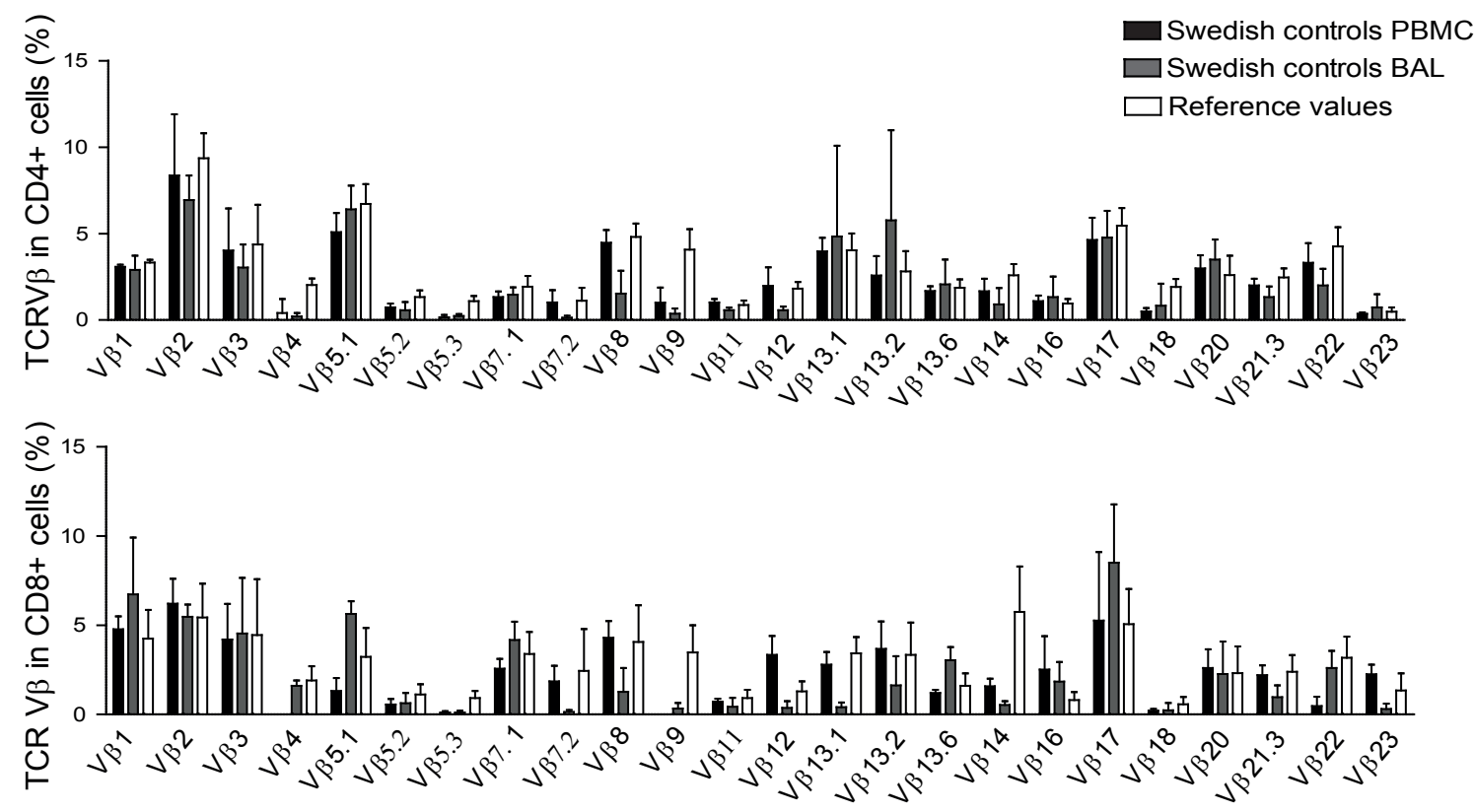

Supplement: Additional file 1 — The healthy control values in this study were compared with reference values. The values of our Swedish cohort is consistent with the reference values, except for Vβ4, Vβ5.3 and Vβ9, which had a very low signal in all samples and Vβ12, which had a higher expression in our CD8+ samples compared to reference values on CD8+ cells. Vβ4, Vβ5.3 and Vβ9 were therefore excluded from all further analyses. Since the normal TCR repertoire in BAL fluid has not been extensively studied, the reference values obtained from whole blood were also used for CD4+ and CD8+ BAL T cells. [file 1471-2466-14-50-S1.pdf]
